# Vaccination of piglets at 2 and 3 weeks of age with Ingelvac PRRSFLEX® EU provides protection against heterologous field challenge in the face of homologous maternally derived antibodies

**DOI:** 10.1186/s40813-016-0037-y

**Published:** 2016-08-14

**Authors:** Gyula Balka, Karla Dreckmann, György Papp, Christian Kraft

**Affiliations:** 1 Department of Pathology, University of Veterinary Medicine, István u. 2, H-1078 Budapest, Hungary; 2 Boehringer Ingelheim Veterinary Research Center GmbH & Co. KG, Bemeroder Str. 31, 30559 Hannover, Germany; 3 Jászapáti 2000 Mezőgazdasági Zrt., H-5130 Jászapáti, Hungary

**Keywords:** PRRS, Vaccination, Modified live virus, Maternally derived antibodies

## Abstract

**Background:**

Due to difficulties in eradicating porcine reproductive and respiratory syndrome (PRRS) linked to biosecurity challenges, transmission of the virus and the lack of efficient DIVA vaccines, successful control of PRRS requires a combination of strict management measures and vaccination of both sows and piglets. The present study aimed to assess the efficacy of a recently developed MLV vaccine (Ingelvac PRRSFLEX® EU) in piglets at 2 and 3-weeks of age in the presence of homologous maternally derived antibodies as the dams were vaccinated with the same vaccine strain (ReproCyc® PRRS EU).

**Methods:**

The study was carried out on a Hungarian farrow to finish farm naturally infected with PRRSv. The study was designed as a blind, placebo controlled side by side trial. ORF5 sequence similarity of the vaccine strain and the resident field strain was 87.8 %. PRRS specific real-time quantitative PCR was performed from serum samples to measure both the viral load and the frequency of virus positive animals.

**Results:**

At the time of the natural infection observed in the control group at 10–12 weeks of age, the number of viraemic animals did not increase significantly in the vaccinated group. To understand the infection dynamics, positive PCR samples with low Ct values were sequenced (ORF5) and the data analysis indicated the circulation of wild type virus in both groups, however wild type virus was only found in non-vaccinated animals.

**Conclusions:**

Our data indicate that piglets vaccinated at as early as 2 weeks of age with Ingelvac PRRSFLEX® EU were protected both in terms of proportion of viraemic animals and viraemia levels. It has to be highlighted that these results were achieved in piglets with high levels of homologous maternally derived antibodies (MDA) at the time of vaccination.

## Background

Porcine reproductive and respiratory syndrome (PRRS) is one of the most widespread, and economically devastating disease in swine industry. It is characterized by reproductive losses in breeding herds, increased mortality in newborn pigs and respiratory disorders in growing pigs [1, 2].

The disease emerged almost at the same time in Europe [3] and North America [4], and since then, it has rapidly spread throughout the world, and become endemic in almost every major swine producing country.

PRRS virus (PRRSV) is a member of the Arteriviridae family within the order of Nidovirales [5]. The relatively small, enveloped virus has a positive-sense single-stranded RNA genome of approximately 15.1 kb in length and encodes 10 ORFs [6–8]. In the last years new ORFs (TF) and −1/−2 programmed ribosomal frameshift signals were discovered in ORF1a, expressing two novel proteins, nsp2TF and nsp2N [9, 10]. Moreover alternative reading frames were identified on the structural protein coding regions: ORF2, ORF5 and most recently on ORF7 coding for GP2a, GP5a and ORF7ap, respectively [11, 12].

Soon after the first isolation, marked genetic differences were identified between these strains and they were classified in two distinct genotypes (Type I, formerly EU, and Type II, formerly NA) [5, 13]. Recent phylogenetic studies performed on Lithuanian, Belarussian and Russian Type I strains revealed unexpectedly high degree of variability within this genotype and led to the definition of four subtypes [14, 15].

The current strategies used to control, or eliminate PRRS require strict management implications including the application of strict biosecurity measures, whole herd depopulation and repopulation, test and removal of seropositive animals, closure of the breeding herd, and vaccination [16].

The marked genetic differences observed among various PRRSV isolates can have a negative effect on the efficacy of modified live vaccines (MLV) [17], however the degree of genetic similarity between the resident strain and the vaccine does not predict the degree of protection conferred after vaccination [18].

Previous studies using Type I MLV and a natural exposure of growing pigs to a field strain of the same genotype reported a reduction of clinical signs, and improved activation of cell mediated immunity in vaccinated animals, but the vaccination did not reduce the incidence of viraemic animals and the levels of viraemia. The duration of viraemia however was shorter in vaccinated piglets [19]. In an other study, performed on PRRSV exposed pregnant sows using commercially available attenuated and farm-specific inactivated vaccine the authors found significantly lower number of viraemic piglets born to sows of vaccinated groups compared to mock-vaccinated ones. Their results indicated that both vaccines could be useful tools in the control of PRRS in the breeding herd [20].

Therapeutic use of a Type II MLV vaccine as an intervention in an acute outbreak was reported to reduce the duration of viral shedding. Also reduced respiratory disease and improved production parameters were recorded when the challenged-vaccinated animals were re-infected with a highly virulent challenge strain [21]. In a recent study the therapeutic, post-infection use of the same Type II MLV was reported to reduce significantly the viral shedding as measured by oral fluid analysis and cumulative PRRSV presence in the air [22].

The objective of the present work was to determine and compare the efficacy of a recently developed MLV vaccine (Ingelvac PRRSFLEX® EU) to a mock (PBS) vaccinated cohort in 2 and 3 weeks old piglets born to sows mass-vaccinated twice with a homologous MLV ReproCyc PRRS® EU and assess the possible interference with homologous maternally derived antibodies with vaccine efficacy.

## Methods

### Animals

This study was conducted in on a commercial, farrow to finish, closed system farm in Hungary. Monitoring over several years showed an ongoing PRRS wild type strain circulation on the farm, confirmed by an actual screening shortly before study initiation. The pre-screening of the herd was performed as a cross sectional ELISA seroprofiling and PCR on serum samples obtained from 80 animals (10 samples of pigs at the age of 2, 4, 6, 8, 10, 12, 14, 16 weeks). The results revealed an ongoing field virus circulation starting in 6-weeks-old animals. Sequences obtained from the study site over time are included in Fig. 4. In total 475 piglets at 2 weeks of age and 551 piglets at 3 weeks of age were included in the study. The batches of piglets were divided into a vaccinated group (Ingelvac PRRSFLEX® EU) and a non-vaccinated control group (246/229 and 351/200 vaccinated/non-vaccinated animals in the 2two- and 3-weeks of age group, respectively). Piglets were vaccinated under the sow and then at 4 weeks of age transferred to one barn that was surrounded by fattening units and farrowing barns. Groups were held in separate rooms and not commingled until the age of 12-weeks of life. The study was blinded for treatment and randomized by farrowing units to prevent cross contamination of non-vaccinated piglets. The piglets originated from sows and gilts that were previously vaccinated with ReproCyc® PRRS EU (Boehringer Ingelheim Vetmedica GmbH, Germany).

### Vaccine strain

The Ingelvac PRRSFLEX® EU vaccine strain (PRRS 94881, full genome Gen Bank accession number KT988004) is attenuated from a field virus first isolated in 2002 from a farm in Germany with clinical symptoms of PRRS. The parental strain belongs to the European Type I, subtype 1 lineage of PRRS viruses. The vaccine strain shared 87.8 % nucleotide identity in the ORF5 gene with the circulating resident wild type PRRSV strain.

### Treatment

Piglets were vaccinated with one dose of Ingelvac PRRSFLEX® EU vaccine with a minimum immunizing dose as indicated on the vaccine label instructions at 2-weeks of age or 3-weeks of age. Control animals were administered one dose of vaccine solvent (PBS) without antigen content. No other vaccinations or treatments were administered to the animals on at least 3 days before and after the PRRS vaccine treatment.

### Sample analysis and assessment of viremia and serology

In each group, 20 % of animals were designated at random as sample animals for blood collection. Blood samples were collected pre-vaccination and then weekly in weeks four to ten after vaccination for the 2 weeks of age groups and weekly in weeks three to nine after vaccination in the 3 weeks of age groups. After drawing, blood samples were allowed to clot at room temperature, were centrifuged and serum harvested. Serum samples were held at −80 °C and for serology and qPCR testing, respectively.

### PRRS serology

For ELISA the IDDEX PRRS X3 test was used following the manufacturer’s instructions (HerdChek* Porcine Reproductive and Respiratory Syndrome Antibody Test Kit X3 – IDEXX Laboratories Inc., Westbrook, ME, USA). Results were reported as negative (ELISA sample to positive [S/P] ratio of < 0.4) or positive (ELISA S/P ratio of ≥ 0.4).

### PRRS serum qPCR

For detection of PRRS virus RNA a validated TaqMan probe based quantitative reverse transcription real time PCR targeting the viral ORF7 was used (bioScreen EVDMC GmbH, Hannover, Germany). Results were reported as negative (n.d.), positive (not quantifiable, <3.0 log10 genome equivalents (GE)/ml) and quantifiable log10 GE/mL. A qPCR result of n.d. (not detected) was assigned a value of 0 log10 GE/mL and a positive qPCR result was assigned a log10 value of 3.0 GE/mL for statistical purposes.

### Sequencing

Serum samples tested positive by qPCR and that exceeded a virus concentration of 3.0 GE/ml were selected for ORF5 sequence analysis. Sequencing of ORF5 was done by amplifying the respective region of the PRRSV genome by PCR directly from the samples, followed by Sanger-sequencing of the purified PCR product according to Balka et al. [23]. The sequencing data obtained was aligned with ORF5 sequences of PRRSV reference strains (i.e. wild type strain circulation on the farm, vaccine strains of commercially available vaccines used on the farm) and subsequent calculation of sequence similarities between the sequences. Sequence analysis was done with CLC Main Workbench v4.1.1.

Phylogenetic analyses were performed using the CLUSTAL X 1.81 software employing IUB DNA weight matrix with 0.5 transition ratio. Bootstrap resampling was carried out on 100 replicate data sets. Phylogenetic trees were plotted with the TREEVIEW (Win32 version 106 1.6.6.) software.

## Results

### Natural challenge

To prove efficacy of a vaccine protection has to be proven by a challenge with virulent wild type PRRS virus. A virulent PRRS virus strain was circulating on the farm before initiation of the study as proven by virus isolation and subsequent sequencing over the past years. Previous cross sectional screening results performed less than 4 months before the start of the study showed an active PRRS circulation in pigs as early as 6 weeks of age, with a peak at 10 weeks of age (data not shown). ORF5 sequence similarity of the field strain and the vaccine strain was 87.8 %. Field challenge in the study animals was also controlled by sequencing of PRRS virus positive blood samples. First signs of a field challenge occurred at 9 weeks of age in non-vaccinated animals. Since the study investigation ended at 12-weeks of age for all animals the peak of field infection might have not been reached at that time.

### Serology

**Fig. 1:**
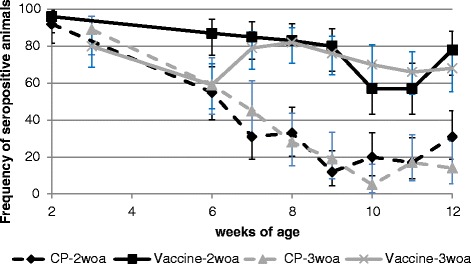
Frequency of seropositive animals vaccinated at 2-weeks of age and 3-weeks of age. Solid curves represent Ingelvac PRRSFLEX EU vaccinated groups, doted curves represent unvaccinated control groups. Vertical bars represent the 95 % confidence interval. CP, control product (mock vaccination); woa, weeks of age

Piglets were tested for PRRS specific antibodies 1 day before vaccination. In the 2-weeks of age group 92 % (48/52; Confidence Interval (CI) 95 %: 81.5–97.9) and 96 % (52/54; CI 95 %: 87.3–99.5) of piglets were seropositive due to maternally derived antibodies in the vaccinated group and the control group, respectively. In the 3-weeks of age group the level of maternal antibodies at study initiation declined to 89 % (39/44; CI 95 %: 75.4–96.2) and 80 % (56/70; CI 95 %: 68.7–88.6) of seropositive piglets in the vaccinated and control group at study inclusion. In the control group the frequency of seropositive pigs continuously and rapidly dropped to 12 % (6/52; CI 95 %: 4.4–23.4) and 5 % (2/42; CI 95 %: 0.6–16.2) until the ninth week of life in the 2-weeks of age group and until the tenth week of life in the 3-weeks of age group, respectively. In contrast, the vaccinated animals remained seropositive throughout the study at a high percentage of animals. In the 2-weeks of age group at least 80 % (43/54; CI 95 %: 66.5–89.4) of animals were tested seropositive until the start of the field challenge. In the initial phase of field challenge the frequency of seropositive animals dropped to 57 % (31/54; CI 95 %: 43.2–70.8), but quickly recovered after 2 weeks. A similar pattern was found in the 3-weeks of age vaccinated group. Again, the frequency of seropositive animals dropped initially to 59 % (40/68; CI 95 %: 46.2–70.6) 3 weeks post vaccination due to declining maternal antibody levels, but recovered to 82 % (58/71; CI 95 %: 70.7–89.9) once vaccination induced antibodies were produced by the animal itself (Fig. 1).

**Fig. 2:**
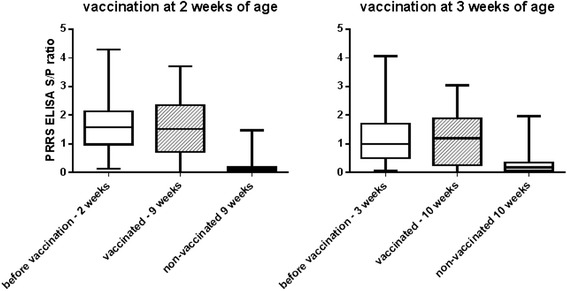
Antibody levels in piglets before and after vaccination. Boxplot of S/P ratios of animals before vaccination and 7-weeks after vaccination. Whiskers represent the min and max ratio

Due to maternally derived antibodies, piglets were positive at vaccination with S/P ratios of 1.62 and 1.16 at 2-weeks and 3-weeks of age, respectively. Vaccinated animals remained this antibody level with S/P ratios of 1.56 at 9-weeks of age in the group vaccinated at 2-weeks of age and 1.14 at 10-weeks of age in the group vaccinated at 3-weeks of age. In contrast, non-vaccinated animals dropped to S/P ratio levels of 0.18 and 0.31 at 9 and 10-weeks of age, respectively (Fig. 2).

### Frequency of viremic animals after vaccination and at field challenge

**Fig. 3:**
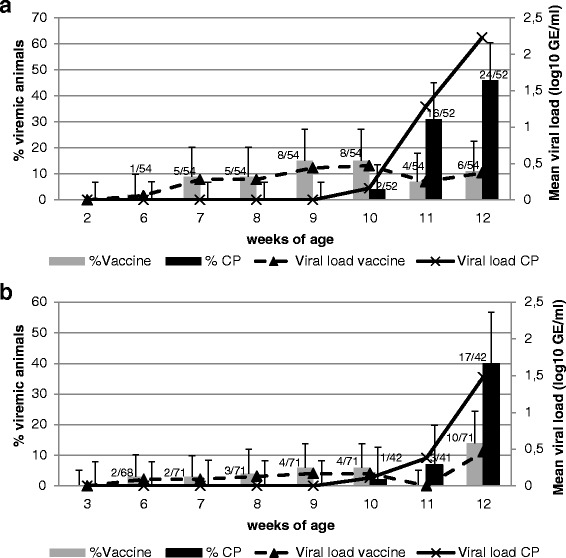
Frequency of qPCR positive animals and viral load in animals vaccinated at **a** 2-weeks of age and **b** 3-weeks of age. Bars represent the per cent of viremic animals (Error indicators represent the upper 95 % confidence interval), curves represent the mean viral load (log_10_ GE/ml) over all animals tested

The vaccinated group showed up to 15 % (8/54 CI 95 %: 6.6–27.1) PRRS positive animals after vaccination in the 2-weeks of age group, while all non-vaccinated animals remained qPCR negative. At the time of field challenge the vaccinated groups both in the 2-weeks of age vaccinated group and the 3-weeks of age vaccinated group stayed at this low level of PRRS positive animals or even declined (Fig. 3). However, upon field challenge the non-vaccinated groups started to rapidly increase the frequency of PRRS positive animals up to 46 % (24/52; CI 95 %: 32.2–60.5) in the 2-weeks of age group and 40 % (17/42; CI 95 %: 25.6–56.7) in the 3-weeks of age group. The difference of affected animals between the vaccinated and control group were highly significant (*p* = 0.0026).

### Detection of serum viral copies after vaccination and at field challenge

Low levels of PRRS viraemia was detected by qPCR in the vaccinated groups shortly after vaccination, while the control groups remained PRRS virus negative in this period (Fig. 3). The maximum mean titer, including all investigated animals within a group, was measured 5 weeks after vaccination at 0.278 log10 GE/mL. All but one positive tested animals at all time points before field challenge showed only non-quantifiable qPCR results. Field challenge occurred at 9 weeks post vaccination as indicated by the appearance of the first PRRSV positive animals in the control group. During the field challenge the mean titer of the vaccinated group peaked at 0.472 log10 GE/mL in the 2-weeks of age group and 0.46 log10 GE/mL in the 3-weeks of age group, while the non-vaccinated animals reached 2.23 log10 GE/mL and 1.48 log10 GE/mL in the 2-weeks and 3-weeks of age groups, respectively. The difference at the end of the study was highly significant for the 2-weeks of age group (*p* < 0.0001) and 3-weeks of age group (*p* = 0.0006).

### Sequence analysis of ORF5 gene

**Fig. 4:**
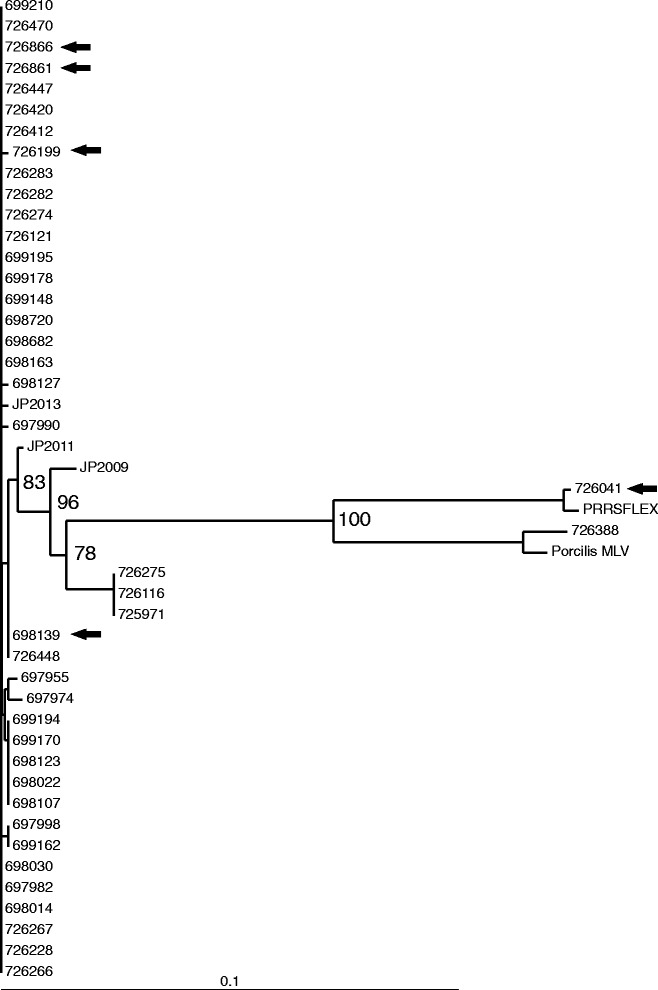
ORF5 sequence analysis of qPCR positive samples. Phylogenetic tree based on the ORF 5 nucleotide sequence data of 42 strains obtained during the study. Bar on the bottom demonstrates the genetic distance. Internal labels represent the bootstrap values of 100 replicates. Arrows indicate the sequences obtained from the vaccinated groups. Jaszapati named strains are resident viruses of the herd identified during the indicated years

In total 42 PRRS positive serum samples originating from 30 animals were subjected to ORF5 sequencing based on quantifiable amounts of genome copies in them (i.e. >3.0 GE/ml). All sequenced samples were originating from the time of wild type PRRSV challenge (Table 1). 37/42 samples belonged to animals of the non-vaccinated control groups, while only five samples were found to be suitable for sequencing from the vaccinated groups (Fig. 4). Complete ORF5 sequences were compared to each other, to the sequences of the resident wild type PRRS strain collected previously, to the Ingelvac PRRSFLEX® EU vaccine strain, and a commercially available MLV strain used previously on the farm. 40/42 strains were identical or very closely related (>98 % similarity) to the resident wild type strain. One control animal carried a derivative strain of a commercial MLV strain (previously used in the farm) with 98.4 % sequence homology, and from one vaccinated animal the Ingelvac PRRSFLEX® EU vaccine-like strain with 99.5 % sequence identity was isolated.

**Table 1:** Group assortment of sequenced isolates

Vaccination at	2-weeks of age		3-weeks of age	
	IVP	CP	IVP	CP
10 weeks of life	1	1	0	1
11 weeks of life	1	8	0	3
12 weeks of life	1	18	2	6

*CP* control product

## Discussion

The present study aimed to assess the field efficacy of a recently developed MLV PRRS vaccine (Ingelvac PRRSFLEX® EU) in piglets at 2 and 3 weeks of age in the presence of homologous maternally derived antibodies. At the time of the field challenge infection, observed in the control group (10–12 weeks of age, WOA), the number of viraemic animals did not increase in the vaccinated group. It has to be highlighted that results were achieved in piglets with high levels of homologous maternally derived antibodies at the time of vaccination since the dams were vaccinated with the same vaccine strain (ReproCyc® PRRS EU).

The protective effect of the MLV against natural challenge is either related to the successful induction of neutralizing antibodies (NAs), and/or boost of the cellular immunity. Previous studies have shown that passive transfer of NAs can protect pregnant sows from viraemia, transplacental shedding and reproductive failure, whereas titres higher that 1:32 can confer sterilizing immunity in growing pigs [24, 25]. The protection however will be less effective in case of heterologous challenge even in the same genotype [17]. The genetic differences however (usually expressed as ORF5 similarities) between the immunizing and the challenging strains cannot predict the degree of the protective immunity conferred [18]. Moreover marked differences have been observed among PRRSV isolates both in terms of their susceptibility to neutralization by NAs induced by other strains and also in their ability to induce NAs that are neutralizing a broad spectrum of isolates [26].

Other authors found that vaccine efficacy in their heterologous challenge model was attributed to the ability of the MLV to boost the cellular immunity by the induction of IFN-γ secreting cells that inversely correlated with the production of IL-10 by PBMCs [27].

Our results indicate proper efficacy of the vaccine strain in terms of proportion of viraemic animals as well as levels of viraemia, however further studies are needed to assess the neutralization ability and spectrum of the NAs induced by Ingelvac PRRSFLEX® EU, and it’s ability to induce specific cellular immunity.

Another critical part of the results was the presence of relative high amounts of homologous maternal antibodies at the time of vaccination. Evidence can be found in the literature proving the negative effect of the MDAs on the antibody responses after vaccination (reviewed in [28, 29]). For example, piglets vaccinated in the presence of MDAs had lower levels of antibody response in case of Aujeszky disease [30], swine influenza virus [31], and *Mycoplasma hyopneumoniae* [32]. On the other hand, the development of the specific cell mediated immunity seems to be less affected or even enhanced by the MDAs. In a recent study piglets vaccinated at 7 days of age in the presence of MDAs against *Mycoplasma hyopneumoniae* showed an earlier cell mediated immunity even though they failed to show vaccine-induced antibody response [33], however results obtained in the case of other pathogens can not be directly adapted to PRRSV infection.

Titre dependent interaction between MDAs and vaccination has been observed by van Woensel et al. [34], who observed high proportion of vaccine takes either at high and low antibody titres, but not at medium titres. The authors explained this observation by the effect of opsonization as an alternative mechanism of PRRSV to enter in the macrophages at high antibody titres. However this Fc receptor based entry route for PRRSV has been questioned lately [35]. However it has to be highlighted, that S/P ratios as measured by ELISA test in our study do not necessarily correspond to antibody titre values and even less to neutralizing antibody levels.

The partial interference of the MDAs with the vaccination cannot be excluded in our case as MDA positive and negative groups were not compared in this study. Certainly, the vaccinated group developed immunity allowing to keep the proportion of the viraemic piglets under 10 % despite the 30–45 % found in the control group.

In our experiment we found that correct timing of the vaccination is more dependent on the presumed natural challenge, than the amount of MDA in the piglets. The vaccine was able to overcome the suppressing effects of the homologous MDA and the time frame between the vaccination and the challenge – observed at 8–10 weeks of age in the control group – was sufficient to develop effective immunity and preventing infection. According to our results, cross sectional seroprofiling and virus detection in the affected age groups is recommended in order to identify the time of the infection and ensure ideal timing for vaccination.

Comparative phylogenetic analyses were conducted after sequencing the ORF5 region of 42 positive samples with sufficient amounts of viral copies (39 from non-vaccinated and five from vaccinated group). 40/42 strains were identical or very closely related (>98 % similarity) to the resident wild type strain. The presence of the wild type virus in the vaccinated group (4 cases) confirmed the infection pressure by the resident virus on the farm. Control and vaccinated groups were located in different rooms in the same barn to prevent the possible transmission of the MLV strain, but also the rooms were randomly distributed in the same building to provide the same environment in terms of virological pressure. One control animal carried a derivative strain of a commercial vaccine strain with ORF5 98.4 % sequence identity, and from one vaccinated animal the Ingelvac PRRSFLEX® EU vaccine strain was isolated.

## Conclusions

Summarizing the data we can conclude that piglets vaccinated at 2 and 3 weeks of age with Ingelvac PRRSFLEX® EU were protected against natural infection both in terms of viraemia levels and proportion of viraemic animals.
